# Arztbegleitete Interhospitaltransporte – eine Analyse aus Schleswig-Holstein

**DOI:** 10.1007/s00063-024-01119-x

**Published:** 2024-03-07

**Authors:** Andrea Köser, Christine Eimer, Maximilian Feth, Ulf Lorenzen, Stephan Seewald, Henrik Lehn, Michael Corzillius, Bjarne Schmalbach, Florian Reifferscheid

**Affiliations:** 1 Klinik für Anästhesiologie und operative Intensivmedizin, Campus Kiel, Universitätsklinikum Schleswig-Holstein, Arnold-Heller-Straße 3, 24105 Kiel, Deutschland; 2 Interdisziplinäre Notaufnahme und Aufnahmestation, Campus Kiel, Universitätsklinikum Schleswig-Holstein, Arnold-Heller-Straße 3, 24105 Kiel, Deutschland; 3 Klinik für Anästhesiologie, Intensivmedizin, Notfallmedizin, Schmerztherapie, Bundeswehrkrankenhaus Ulm, Oberer Eselsberg 40, 89081 Ulm, Deutschland; 4 Institut für Rettungs- und Notfallmedizin, Universitätsklinikum Schleswig-Holstein, Arnold-Heller-Straße 3, Haus 808, 24105 Kiel, Deutschland; 5 Berufsfeuerwehr Kiel, Westring 325, 24116 Kiel, Deutschland; 6 DRF Stiftung Luftrettung gAG, Rita-Maiburg-Straße 2, 70794 Filderstadt, Deutschland

**Keywords:** Sekundärverlegung, Intensivtransport, ITW, Notfallverlegung, Intensivpflichtige Patienten, Emergency patients, Interhospital critical care transport, Critically ill patients, Intensive care unit, Emergency transfer

## Abstract

**Hintergrund:**

Durch Veränderungen in der Krankenhauslandschaft nehmen Interhospitaltransporte (IHT) einen immer höheren Stellenwert im Rettungsdienst ein. Durch die Spannbreite der Anforderungen hinsichtlich Personals und Rettungsmittel bedürfen diese Transporte einer sorgfältigen Einsatzplanung.

**Zielsetzung:**

Um den Status quo zu untersuchen, führten wir eine Analyse im Versorgungsbereich der Integrierten Regionalleitstelle Mitte (IRLS-Mitte) in Schleswig-Holstein durch.

**Material und Methoden:**

Im Zeitraum vom 01.10.2021 bis 30.09.2022 wurden arztbegleitete IHT analysiert.

**Ergebnisse:**

Von insgesamt 158.823 dokumentierten Einsätzen der ILRS-Mitte konnten 2264 (1,4 %) Datensätze als arztbegleitete IHT identifiziert und eingeschlossen werden. Es erfolgten 1389 Transporte (61,4 %) durch spezialisierte Rettungsmittel, sowie 875 (38,6 %) durch Rettungsmittel der Primärversorgung. Der Einsatz von Primärrettungsmittel erfolgte schwerpunktmäßig bei zeitkritischen Verlegungen und außerhalb der Dienstzeiten des Intensivtransportwagens (ITW)/Verlegungseinsatzfahrzeugs (VEF), 21,2 % erfolgten luftgebunden. Bei 43,1 % aller Transporte handelte es sich um Aufwärtsverlegungen.

**Diskussion:**

Arztbegleitete IHT nehmen einen relevanten Anteil am Einsatzspektrum des Rettungsdiensts ein und betreffen sowohl Rettungsmittel der Primärversorgung als auch spezialisierte Rettungsmittel. Außerhalb der Vorhaltezeiten von ITW/VEF übernehmen Notarzteinsatzfahrzeuge (NEF) und Rettungstransporthubschrauber (RTH) vorrangig die Transporte. Für die nächtlichen Notfalltransporte ist aufgrund des Zeitvorteils eine Ausweitung der luftgebundenen Verlegungskapazitäten zu erwägen. Für weniger dringliche Transporte könnte eine Anpassung der Kapazitäten spezialisierter bodengebundener Rettungsmittel in Schleswig-Holstein sinnvoll sein.

## Abstract (en)

**Background:**

The need for interhospital transport (IHT) of intensive care patients is increasing due to changes in the hospital environment. Interhospital transports are challenging and require careful operational planning of personnel and rescue vehicles.

**Objective:**

To investigate the need for IHT, an analysis was conducted in the service area of the emergency medical service central dispatch center (IRLS) in Schleswig-Holstein.

**Material and methods:**

Emergency physician-assisted IHT were analyzed in the period from 01.10.2021 to 30.09.2022.

**Results:**

Of a total of 158,823 documented IRLS missions, 2264 (1.4%) records could be identified and included as IHT: 1389 IHT (61.4%) were managed by specialized ambulances, 875 (38.6%) by primary care ambulances. Primary care ambulances were mainly used for time-critical transfers and outside the duty hours of the intensive care ambulances, 21.2 % were by air. Of all IHT, 43.1% were required to hospitals with a higher level of medical care.

**Conclusion:**

Emergency physician-assisted IHT are a relevant part of the emergency service’s operational spectrum and concern both primary care and specialized rescue vehicles. A relevant number of urgent IHT were recorded outside the duty hours of the intensive care ambulances. For emergency transports during nighttime, an expansion of air-based transfer capacities should be considered due to the time advantage. For less urgent IHT, an adjustment of the capacities of specialized ground-based vehicles in Schleswig-Holstein seems reasonable.

## Einleitung

Mit der Reform der Notfallversorgung und der anstehenden Krankenhausstrukturreform [1] führen die Veränderungen der Krankenhauslandschaft und der Fachkräftemängel zu einer kontinuierlich steigenden Zahl an Interhospitaltransporten (IHT; [2, 3]). Um diesen Anforderungen besser begegnen zu können, wurde in Schleswig-Holstein im Jahr 2021 eine Koordinierungsstelle für Sekundärtransporte aufgebaut, um die Transporte möglichst bedarfsgerecht zu disponieren.

## Hintergrund

Arztbegleitete IHT werden abhängig von der Erkrankungsschwere des Patienten mit speziellen Rettungsmitteln durchgeführt [4]. In Schleswig-Holstein stehen hierfür sog. Intensivtransportwagen (ITW) bzw. auch Verlegungseinsatzfahrzeuge (VEF) zur Verfügung, die ähnlich einem Notarzteinsatzfahrzeug (NEF), mit einem Notarzt/Notärztin und einem Notfallsanitäter/Notfallsanitäterin und einer erweiterten medizinischen Ausrüstung ausgestattet sind.

Die IHT werden tagsüber bei der Koordinierungsstelle für Sekundärtransporte (KOST-SH) mittels einer standardisierten Anmeldung angefordert. In diesem Rahmen werden die Krankengeschichte, der Bedarf der medizinischen Versorgung und das erforderliche Equipment erfasst. Anhand der Anmeldedaten und der Transportdringlichkeit erfolgt die Disposition des Rettungsmittels durch geschultes, nichtärztliches Personal. Eine ärztliche Abklärung erfolgt nur im Einzelfall.

Im untersuchten Einsatzgebiet stehen seit dem Jahr 2021 2 ITW (Montag bis Freitag, Kiel 8.00 bis 18.00 Uhr, Lübeck 7.00 bis 19.00 Uhr) und 4 VEF (VEF Flensburg 24/7, VEF Kiel und Itzehoe Montag bis Sonntag 7.00 bis 19.00 Uhr, VEF Lübeck Montag–Freitag 7–19 Uhr) zur Verfügung. In den Nachtstunden werden IHT mithilfe der NEF bzw. luftgebunden per Rettungstransporthubschrauber (RTH) geleistet. In Schleswig-Holstein stehen derzeit ein 24 h-RTH sowie 2 RTH im Tagesbetrieb zur Verfügung. In besonderen Fällen übernimmt ein Offshore-Hubschrauber auch Einsätze in Kooperation mit der IRLS, dieser steht aber grundsätzlich für die Versorgung der Offshore-Windenergieanlagen in der Nordsee zur Verfügung. Die Disposition der Sekundärtransporte erfolgt tagsüber zentral über die KOST-SH, nachts übernehmen die örtlich zuständigen Leitstellen die Disposition.

## Fragestellung

Das Ziel dieser Studie war es, die IHT hinsichtlich Einsatzhäufigkeit, Transportdringlichkeit, die tageszeitliche Verteilung der Transporte und ihre geografische Reichweite zwischen den Start- und Zielkliniken zu evaluieren.

### Studiendesign und Untersuchungsmethoden

In Zusammenarbeit mit der Berufsfeuerwehr Kiel wurden in einer retrospektiven Analyse alle durch die KOST-SH (landesweit) sowie nachts durch die IRLS-Mitte (Stadt Kiel, Landkreis Plön, Landkreis Rendsburg-Eckernförde) disponierten Rettungsdiensteinsätze im Zeitraum vom 01.10.2020 bis zum 30.09.2021 ausgewertet. Es wurden alle arztbegleiteten IHT in die Studie eingeschlossen, nach Rettungsmittel sortiert und hinsichtlich ihrer geografischen Verteilung der Start- und Zielkliniken und der Dringlichkeit (Dringlichkeit 1 = Eintreffen Quellklinik < 30 min ab Anmeldung; Dringlichkeit 2 = Transportbeginn < 2 h; Dringlichkeit 3 = planbarer Transport) analysiert. Einige Einsätze wurden ohne Angabe einer Dringlichkeit disponiert, sodass „keine Angabe“ (k. A.) vermerkt wurde. Außerdem wurden die tageszeitliche Verteilung, die Transportzeiten (Anfahrt, Übernahme, Patiententransport, Einsatzdauer) und die Transporte entsprechend der Versorgungsstufe der beteiligten Kliniken in „Aufwärtsverlegungen“ zu einer Klinik einer höheren Versorgungsstufe bzw. in „Abwärtsverlegungen“ zu einer Klinik einer niedrigeren Versorgungsstufe und Horizontalverlegungen zwischen Kliniken der gleichen Versorgungsstufe eingeteilt.

### Statistik

Sämtliche Auswertungen und Abbildungen wurden mittels „R“, (Version 4.2.3 R Core Team, Free Software Foundation’s GNU projekt, Vienna, Austria) und Microsoft Excel 2022 (Excel, Microsoft Corporation, Redmont, WA, USA) erstellt. Kategoriale Variablen sind als absolute und relative Häufigkeiten (%), ordinal skalierte und metrische Variablen als Mittelwert und Standardabweichung (Normalverteilung) oder Median mit Interquartilsabstand (nichtparametrische Verteilung) dargestellt.

### Ethik

Das vorliegende Studienvorhaben wurde von der Ethikkommission der Medizinischen Fakultät der Christian-Albrechts-Universität zu Kiel genehmigt (AZ: D 544/21).

## Ergebnisse

**Abb. 1:**
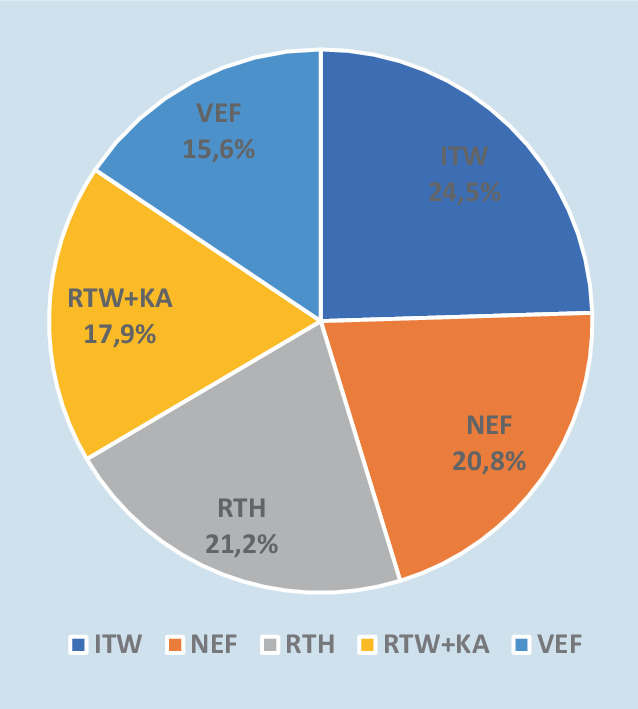
Darstellung der arztbegleiteten Interhospitalverlegungen aufgeteilt nach den verfügbaren Rettungsmitteln

Von insgesamt 158.823 dokumentierten Einsätzen der IRLS-Mitte konnten 2264 (1,4 %) arztbegleitete IHT in die Analyse eingeschlossen werden. 875 (38,6 %) Transporte erfolgten durch Rettungsmittel der Primärversorgung wie NEF (470; 20,8 %), RTW plus Klinikarzt (RTW plus KA; 405; 17,9 %) sowie RTH (481; 21,2 %). 1389 Transporte (61,4 %) erfolgten durch die spezialisierten Rettungsmittel ITW (555; 24,5 %) und VEF (353; 15,6 %; Abb. 1).

### Dringlichkeit

Es erfüllten 1093 (48,2 %) Transportanmeldungen die Kriterien der Dringlichkeitsstufe 1, diese Transporte wurden in absteigender Häufigkeit von RTW plus KA (32,6 %), NEF (31,0 %), RTH (21,2 %), VEF (9,7 %) und ITW (5,5 %) abgearbeitet. Es wurden 380 (16,8 %) Transportanmeldungen mit der Dringlichkeitsstufe 2 angemeldet, davon erfolgten 146 (38,4 %) Transporte mittels RTH, 96 (25,3 %) mittels VEF, 73 (19,2 %) mittels ITW und 42 (11 %) mittels NEF bzw. RTW plus KA (23; 6 %). Mit der Dringlichkeitsstufe 3 wurden insgesamt 692 (30,6 %) Transporte angemeldet, davon wurden 401 (57,9 %) mit dem ITW, 136 (19,6 %) mittels VEF, 90 (13,0 %) mittels RTH, 42 (6 %) mit dem NEF und 23 (3,3 %) mit RTW plus KA transportiert. 99 (4,4 %) Transporte wurden ohne Angabe einer Dringlichkeit angefordert (s. Tab. 1).

**Tab. 1:** Verteilung der Dringlichkeit der Einsätze, Prozentangaben in Bezug auf die jeweilige Rettungsmittelart

Dringlichkeit	ITW (%)	NEF (%)	RTH (%)	RTW + KA (%)	VEF (%)	Gesamt (%)
1 (< 0,5 h)	60 (5,5)	339 (31)	232 (21,2)	356 (32,6)	106 (9,7)	1093 (48,2)
2 (< 2 h)	73 (19,2)	42 (11)	146 (38,4)	23 (6)	96 (25,3)	380 (16,8)
3 (disponibel)	401 (57,9)	42 (6)	90 (13)	23 (3,3)	136 (19,6)	692 (30,6)
k. A.	21 (21,2)	47 (47,5)	13 (13,1)	3 (3)	15 (15,1)	99 (4,4)
Gesamt	555	470	481	405	353	2264

**Tab. 2:** Darstellung der Dringlichkeit in Bezug auf die Tages- und Nachtzeit, Prozentangaben in Bezug zur Tageszeit

Dringlichkeit	Tag (8–20 Uhr) (%)	Nacht (20–8 Uhr) (%)
1 (< 0,5 h)	806 (44,2)	287 (65,4)
2 (< 2 h)	304 (16,7)	76 (17,3)
3 (disponibel)	638 (35,0)	54 (12,3)
Keine Angabe	77 (4,2)	22 (5,0)
Gesamt	1825	439

### Auf- und Abwärtsverlegungen

Aufwärtsverlegungen waren der häufigste Grund zur Alarmierung eines Sekundärtransports (975; 43,1 %). Bei einer Aufwärtsverlegung handelt es sich um eine Verlegung in eine höher qualifizierte Versorgungseinheit, z. B. von einem Schwerpunktversorger in eine Klinik der Maximalversorgung oder aber von der Notaufnahme des einen auf die Intensivstation des anderen Krankenhauses [5]. Die Transporte erfolgten in absteigender Häufigkeit mittels NEF (360; 36,9 %), RTH (271; 27,8 %), VEF (152; 15,6 %), ITW (148; 15,2 %) und RTW plus KA (44; 4,5 %). Es wurden 625 (27,6 %) Abwärtsverlegungen in eine Klinik einer niedrigeren Versorgungsstufe bei der Leitstelle angemeldet, diese wurden hauptsächlich durch den ITW (345; 55,2 %), VEF (159; 25,4 %), danach folgend von NEF (77; 12,3 %), RTH (32; 5,1 %) und RTW plus KA (12; 1,9 %) durchgeführt. Horizontalverlegungen, z. B. eine Verlegung zwischen Kliniken der gleichen Versorgungsstufe, waren im Studienzeitraum in 593 (26,19 %) Einsätzen erforderlich, diese wurden hauptsächlich durch RTW plus KA (344; 58,0 %); RTH (145; 24,4 %); ITW (52; 8,7 %), VEF (33; 5,5 %) und NEF (19; 3,2 %) vorgenommen. 344 Transporte mit RTW plus KA wiesen eine sehr kurze Transportdistanz von 0,3 km (Median) auf, hierbei handelt es sich mutmaßlich um innerklinische Transporte auf dem Campus des Universitätsklinikums in Kiel (s. Abb. 4). In 71 (3,1 %) Einsätzen erfolgte hinsichtlich der Transportrichtung keine Angabe (s. Tab. 3).

**Tab. 3:** Transportrichtung der Verlegungen in Bezug auf die Versorgungsstufen von Start- und Zielkrankenhaus; Prozentangaben in Bezug auf die jeweilige Rettungsmittelart

Transportrichtung	ITW (%)	NEF (%)	RTH (%)	RTW + KA (%)	VEF (%)	Gesamt (%)
Aufwärtsverlegungen	148 (15,2)	360 (36,9)	271 (27,8)	44 (4,5)	152 (15,6)	975 (43,1)
Abwärtsverlegungen	345 (55,2)	77 (12,3)	32 (5,1)	12 (1,9)	159 (25,4)	625 (27,6)
Horizontalverlegungen	52 (8,7)	19 (3,2)	145 (24,4)	344 (58)	33 (5,5)	593 (26,2)
k. A.	10 (14,1)	14 (19,7)	33 (46,5)	5 (7)	9 (12,6)	71 (3,1)

### Verteilung der Einsätze nach Wochentagen und Tag/Nacht

**Abb. 2:**
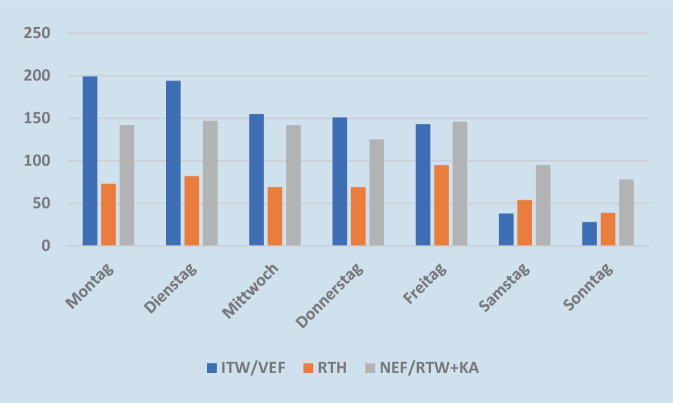
Transporte aufgeteilt nach Rettungsmittel anhand ihrer Einsatzfrequenz an den einzelnen Wochentagen. x‑Achse: Aufteilung nach Wochentagen; y‑Achse: Darstellung der Einsatzfrequenz

Der Großteil der Einsätze erfolgte von Montag bis Freitag, 14,7 % aller Transporte waren an den Wochenenden erforderlich. 80,6 % der Einsätze wurden tagsüber zwischen 8.00 und 20.00 Uhr und 19,4 % nachts zwischen 20.00 und 8.00 Uhr durchgeführt. Tagsüber waren 44,2 % in der Dringlichkeit 1, 16,7 % in der Dringlichkeit 2 und 35 % in der Dringlichkeit 3, 4,2 % sonstige. In der Nacht waren 65,4 % Transporte der Dringlichkeit 1, 17,3 % Dringlichkeit 2, 12,3 % Dringlichkeit 3 und 5,0 % keiner Dringlichkeit zugeordnet (s. Tab. 2 und 4 und Abb. 2 und 3).

**Tab. 4:** Darstellung der Einsatzanzahlen der jeweiligen Rettungsmittel in Bezug zum Wochentag

Wochentag	ITW	NEF	RTH	RTW + KA	VEF	Gesamt (%)
Montag	127	75	73	67	72	414 (18,3)
Dienstag	132	64	82	83	62	423 (18,7)
Mittwoch	91	71	69	71	64	366 (16,2)
Donnerstag	103	57	69	68	48	345 (15,2)
Freitag	95	74	95	72	48	384 (17,0)
Samstag	4	70	54	25	34	187 (8,3)
Sonntag	3	59	39	19	25	145 (6,4)

**Abb. 3:**
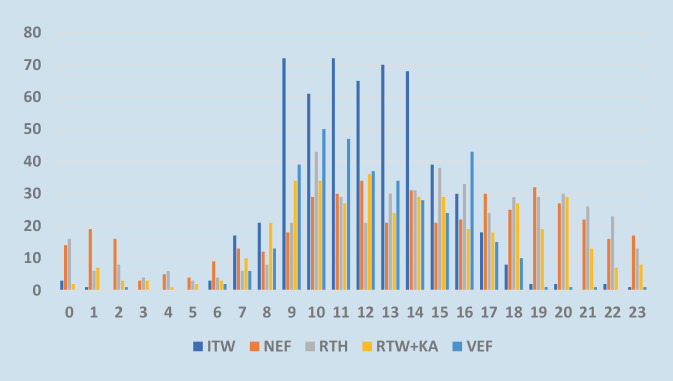
Tageszeitliche Verteilung der Transporte dargestellt anhand der Einsatzfrequenz über 24 h. x‑Achse: Aufteilung in Stunden anhand der Uhrzeit von 0.00 bis 23.00 Uhr; y‑Achse: Darstellung der Einsatzfrequenz

### Transportintervalle und Distanzen

**Abb. 4:**
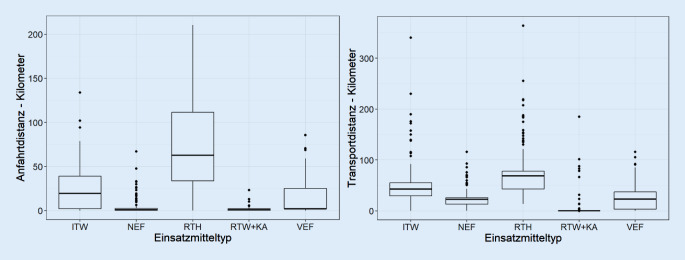
Die Boxplots zeigen die Verteilung der Distanzen der jeweiligen Rettungsmittel. Boxplot: Median, horizontaler Marker innerhalb der Box; „range“ der Box von der 25. bis zur 75. Perzentile und Darstellung der Min- bzw. Maximalausprägungen

Die Anfahrtsdistanz bis zur Startklinik betrug beim ITW im Median 19,6 km (IQR 36,8), NEF 0,9 km (IQR 2,2), RTH 62,9 km (IQR 77,6), RTW plus KA 1,5 km (IQR 1,8) und VEF 2,1 km (IQR 23,3). Die Transportdistanz betrug beim ITW im Median 42,8 km (IQR 25,5), NEF 23 km (IQR 12,1), RTH 68,8 km (IQR 34,7), RTW plus KA 0,3 km (IQR 0,02) und VEF 23,1 km (IQR 34,0). Somit wird deutlich, dass der RTH die größten Anfahrts- und Transportdistanzen zurücklegt. Gleiches gilt für den ITW, der ebenfalls längere Anfahrts- und Transportstrecken aufweist (s. Abb. 4).

**Abb. 5:**
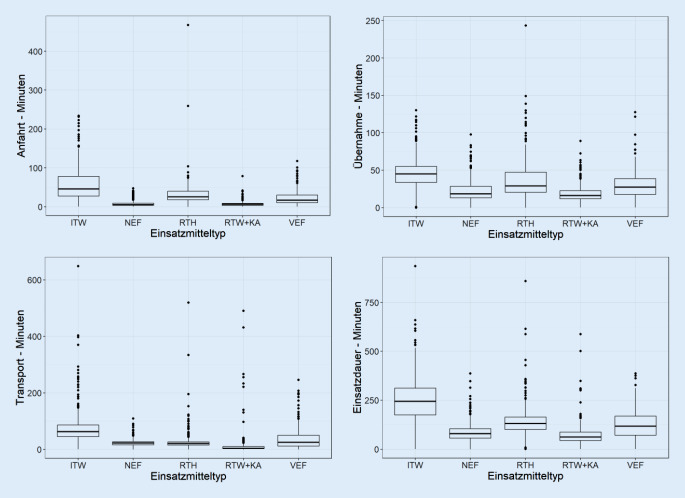
Die Boxplots zeigen die Verteilung des Zeitaufwands der jeweiligen Rettungsmittel. Boxplot: Median, horizontaler Marker innerhalb der Box; „range“ der Box von der 25. bis zur 75. Perzentile und Darstellung der Min- bzw. Maximalausprägungen

Die Übernahmezeit (Status 4 bis Status 7) betrug im Median beim ITW 45,1 min (IQR 21,6), beim NEF 18,5 min (IQR 15,6), beim RTH 29 min (IQR 26,9), beim RTW plus KA 16,1 min (IQR 10,9) und beim VEF 27,3 min (IQR 21,3). Die Zeiten zur Übernahme waren bei ITW, RTH und VEF länger als im Vergleich zu den Primärrettungsmitteln. Die Gesamtzeit des Einsatzes war bei Nutzung des ITWs am längsten (s. Abb 5).

## Diskussion

Wir führten eine Analyse der IHT hinsichtlich der Anzahl der eingesetzten Rettungsmittel, der Dringlichkeit und der tageszeitlichen und geografischen Verteilung der Start- und Zielkliniken durch. Die Spannbreite der Einsätze reichte dabei von intensivmedizinischen Aufwärtsverlegungen bis hin zu Abwärtsverlegungen in eine Rehabilitationseinrichtung. Da die Vorgaben für einen Transport der Dringlichkeitsstufe 1 bei < 30 min liegen, erfolgten diese Transporte vorwiegend mittels RTH oder NEF aus der Primärversorgung, da für die spezialisierten Verlegungsfahrzeuge, wie VEF und ITW mit oft längerer Anfahrt bedingt durch weniger bzw. entferntere Standorte, dies in der Zeitvorgabe schwierig einzuhalten ist. Für Transporte der Dringlichkeit 2 (Transport innerhalb von 2 h) wurden vorwiegend RTH und VEF eingesetzt. Transporte der Dringlichkeit 3 wurden vor allem mit den Sekundärrettungsmitteln ITW und VEF vorgenommen. Da es sich hier um planbare Verlegungen handelte, konnten diese Transporte problemlos auch mit entfernter stationierten Fahrzeugen durchgeführt werden. Anhand der Analyse von Distanzen und Versorgungsstufen zeigt sich, dass der ITW vor allem für Abwärtsverlegungen eingesetzt wurde. Hingegen wurden Aufwärtsverlegungen, die in der Regel zeitkritisch erfolgten, im untersuchten Kollektiv meistens durch NEF oder RTH durchgeführt. In 30,8 % erfolgte die Aufwärtsverlegung mit den Rettungsmitteln ITW und VEF. Die Kombination aus RTW plus KA wurde fast ausschließlich für kurzstreckige Transporte eingesetzt. Hier ist zu diskutieren, inwiefern Transporte innerhalb eines Klinikcampus zu den Aufgaben des Rettungsdiensts gehören. In der untersuchten Stichprobe wurden sie jedoch durch die IRLS-Mitte disponiert und mit öffentlichen Rettungsmitteln durchgeführt. Einige der Horizontal- bzw. Abwärtsverlegungen wurden durch die Rettungsmittel der Primärversorgung RTH (abwärts: 5,1 %; horizontal: 24,5 %) und NEF (abwärts: 12,3 %; horizontal: 3,2 %) geleistet. Dies ist unter anderem dadurch zu erklären, dass durch die Schleswig-Holstein-spezifische Situation der Versorgung der Nordseeinseln und den bevölkerungsärmeren westlichen Teil des Bundeslands häufig diese Transportmittel als einziges zur Verfügung stehen.

### Einsatzplanung

Wiegersma et al. konnten zeigen, dass schwerwiegende Komplikationen deutlich häufiger auftraten, wenn Rettungsmittel aus dem Regelrettungsdienst für die IHT verwendet werden [6], hier wurde der nichtärztlich begleitete IHT betrachtet. Die Komplikationsrate war bei Verwendung von speziell ausgerüsteten Intensivverlegungsfahrzeugen und entsprechend geschultem Personal geringer. Ligtenberg et al. zeigten in ihrer Untersuchung von 100 IHT, dass in etwa einem Drittel aller Transporte Komplikationen auftraten, von diesen Zwischenfällen hätte bei besserer Vorbereitung der Transporte ein Großteil vermieden werden können [7]. Trotz des Vergleichs zum nichtärztlich begleiteten Interhospitaltransport sprechen diese Zahlen deutlich für die Notwendigkeit einer indikationsgerechten Einsatzplanung seitens der Leitstelle. Strauch et al. beziffern die Rate an unvorhergesehen Zwischenfällen während des Transports mit 6,4 % [8]. Auch seitens der DIVI wurde diese Problematik thematisiert, sodass Empfehlungen für die ärztliche und nichtärztliche Weiterbildung herausgegeben wurden und ein 20-stündiges Curriculum zur Vorbereitung des Personals auf IHT angeboten wird [9, 10]. In Schleswig-Holstein ist die Teilnahme an diesem Kurs für das ITW-Personal, jedoch nicht für das Personal des VEF, verpflichtend.

Der Bedarf einer indikationsgerechten Einsatzplanung beinhaltet auch, solche Transporte herauszufiltern, die die Kriterien eines arztbegleiteten Transports oder eines bestimmten Transportmittels nicht erfüllen. Eine ärztliche Indikationsprüfung vor Disposition des angeforderten Rettungsmittels ist in Deutschland nur in wenigen Rettungsdienstbereichen üblich [11, 12]. Allerdings konnten Schroeder et al. zeigen, dass durch eine entsprechende Überprüfung die Anzahl der arztbegleiteten Transporte gesenkt werden konnte, viele Verlegungen konnten auch mit telemedizinischer Unterstützung begleitet werden [13, 14]. Dies ist auch im Kontext des im ländlichen Raum bestehenden Mangels an Notärzten ein wichtiger Baustein für die Versorgung der Patienten.

### Transportmittel

Die Dringlichkeit der Verlegung bestimmt maßgeblich das Transportmittel. Dies deckt sich mit Daten von Roessler et al., der Zeitvorteil war in der Studie aus Niedersachen in 58,3 % der Fälle entscheidend für die Disposition des Rettungsmittels [15]. Darüber hinaus spielt auch die geografische Lage der Quellklinik bei der Auswahl der Fahrzeuge eine wichtige Rolle. In unserer Analyse zeigt sich, dass mit Ausnahme des Hubschraubers deutlich längere Bereitstellungszeiten der spezialisierten Rettungsmittel (ITW, VEF) erforderlich sind. Der ITW erledigte im Studienzeitraum 555 Transporte, das VEF 353. Dabei wurden mit dem VEF mehr dringliche Verlegungen vorgenommen als mit dem ITW, dies gleicht den Daten von Skazel et al. [16]. In Schleswig-Holstein wurden VEF erst vor wenigen Jahren, ähnlich wie z. B. in Bayern, eingeführt und erweitern das Spektrum arztbesetzter Fahrzeuge, was nicht in allen 16 Bundesländern vorgesehen ist. Gerade bei langen Anfahrtswegen kommt der RTH als Rettungsmittel der Wahl in Betracht. Hinsichtlich ITW und VEF zeigt sich, dass diese Transportmittel im Vergleich die längste Anfahrts- und Transportzeit benötigen und gerade der ITW eine signifikant längere Einsatzdauer im Vergleich zu den anderen Rettungsmitteln aufweist (s. Abb. 4). Die Länge der Einsatzdauer mag dabei auch durch die zeitintensive, komplexe Verladung der Patienten und des ggf. umfangreichen medizinischen Equipments in den RTH bzw. ITW begründet sein, so zeigen sich bei den spezialisierten Einsatzmitteln auch signifikant längere Übernahmezeiten als im Vergleich zu NEF und RTW plus KA (s. Abb. 4). Aus der vorliegenden Analyse wird deutlich, dass Primärrettungsmittel vor allem bei Verlegungen mit kurzer Anfahrts- und kurzer Transportdistanz eingesetzt werden. Transportbegleitungen durch einen Primärnotarzt sollten vermieden werden, da dieser in seinem Einsatzgebiet für die Notfallrettung dann nicht zur Verfügung steht. In diesem Gedanken werden Verlegungen über längere Strecken häufiger mit Sekundärrettungsmitteln durchgeführt, wobei die längsten Distanzen mit dem RTH überwunden werden. Dies zeigt, dass seitens der Leitstelle gerade bei längeren Wegstrecken auf eine zweckgerichtete Disposition der Rettungsmittel Wert gelegt wird.

### Dringlichkeit

Im vorliegenden Einsatzkollektiv wurden 48,2 % aller IHT mit der Dringlichkeit 1 disponiert und vorwiegend mittels NEF und RTW plus KA geleistet. Daraus geht hervor, dass Primärrettungsmittel vor allem bei Verlegungen mit kurzer Anfahrts- und kurzer Transportstrecke eingesetzt werden, sodass das NEF über den Zeitraum des IHT für die Notfallrettung nicht zur Verfügung steht. Transporte der Dringlichkeit 1 wurden in 21,2 % mit dem RTH und lediglich in 5,5 % mit dem ITW transportiert. Dies mag einerseits aus geografischen Gründen der Fall sein (z. B. lange Anfahrtszeit), andererseits könnten hierfür auch die Dienstzeiten des ITW ursächlich sein, die meist auf die Wochentage Montag bis Freitag und die Uhrzeit zwischen 8.00 und 18.00 Uhr begrenzt waren. Diese Annahme wird auch dadurch unterstützt, dass von insgesamt 1093 Transporten der Dringlichkeit 1, 287 (26,3 %) in den Nachtstunden angefordert wurden, in denen der ITW außer Dienst ist. Da bei der Dringlichkeit 1 der Zeitfaktor eine entscheidende Rolle spielt und zwei Drittel der nächtlichen Verlegungen in dieser Kategorie lagen, ist zu überlegen, die nächtliche Abdeckung der Luftrettung für Akutverlegungen zu erweitern, da ein bodengebundenes Netz aus VEF diese Dringlichkeitsstufe nur eingeschränkt bedienen kann. Für die Transporte der Dringlichkeit 2 könnte eine bessere geografische Abdeckung der spezialisierten bodengebundenen Rettungsmittel sinnvoll sein, um die Anfahrtsdistanzen zu verkürzen.

### Limitationen

Im Rahmen des für die Studie verfügbaren Datensets konnten keine Informationen über den jeweiligen Gesundheitszustand der Patienten gewonnen werden, sodass auch der Bedarf an medizinischen Maßnahmen (Beatmung, Medikamente etc.) nicht evaluiert werden konnte. Diese Thematik sollte in weiteren Studien stärker berücksichtigt werden.

## Schlussfolgerung und Fazit

Auf den IHT spezialisierte Rettungsmittel sind gerade für den Transport kritisch kranker Patienten erforderlich, aufgrund der geringen Einsatzmittelanzahl und deren geografischer Verteilung ist bei akuten Notfallverlegungen der Dringlichkeit 1 der luftgebundene Transport deutlich überlegen. Eine stärkere Inanspruchnahme der Luftrettung, vor allem in den Nachtstunden, scheint naheliegend. Für Transporte der Dringlichkeit 2 und 3 kann eine Erweiterung der bodengebundenen Rettungsmittel eine gute Ergänzung sein, um die Fahrzeuge aus der Primärversorgung zu entlasten. Im Zuge der Krankenhausstrukturreform und einer damit einhergehenden Zusammenlegung von Klinikstandorten wird die Erweiterung der Transportkapazitäten, sowohl luft- als auch bodengebunden, für die Patientenversorgung zunehmend eine Schlüsselrolle bilden. Daher erscheint ein Ausbau der Strukturen und eine zentrale Koordinierungsstelle notwendig und sinnvoll, um nach standardisierter Abfrage ressourcengerecht das optimale Rettungsmittel einzusetzen.

## Fazit für die Praxis

- Vor Transportbeginn sollten Art und Umfang des Transports durch einen auf Interhospitaltransporte (IHT) geschulten Leitstellenmitarbeiter/-in anhand konkreter Algorithmen überprüft und das jeweils geeignete Rettungsmittel disponiert werden.
- Bei Transporten der Dringlichkeit 1 ist die Möglichkeit eines luftgebundenen Transports zu prüfen.
- Mittels Verlegungseinsatzfahrzeugen (VEF) können viele weniger komplexe Verlegungen zeiteffektiv erfolgen.
- Für die adäquate Versorgung und den Transport des intensivpflichtigen Patienten ist eine Verfügbarkeit von spezialisierten Rettungsmitteln auch in den Abend- und Nachtstunden zu prüfen.
- Möglicherweise können zukünftig viele IHT mit telemedizinischer Unterstützung begleitet und die Auslastung spezialisierter Rettungsmittel reduziert werden.
